# Androgenic properties of the dietary supplement 5α-hydroxy-laxogenin

**DOI:** 10.1007/s00204-022-03283-5

**Published:** 2022-03-28

**Authors:** Carolin Beer, Annekathrin M. Keiler

**Affiliations:** 1 Institute of Doping Analysis and Sports Biochemistry Dresden, Kreischa, Germany; 2 Environmental Monitoring and Endocrinology, Faculty of Biology, Dresden, Germany

**Keywords:** 5α-hydroxy-laxogenin, PC3(AR)_2_ cells, Yeast androgen screen, Androgen receptor, Dietary supplements

## Abstract

Dietary supplements sold for anabolic benefits or performance enhancement often contain substances, which are non-approved and might lack quality controls. With regard to athletes, the inclusion of substances or methods in the prohibited list of the World Anti-Doping Agency is based on medical or scientific evidence. 5α-hydroxy-laxogenin is a synthetic spirostane-type steroid, which is contained in dietary supplements and advertised as anabolic agent. To date, evidence is missing on anabolic or androgenic activity of 5α-hydroxy-laxogenin. We investigated its androgenic potential in two in vitro bioassays. While no activity was observed in the yeast androgen screen, 5α-hydroxy-laxogenin was able to trans-activate the androgen receptor in human prostate cells in a dose-dependent manner. Interestingly, a biphasic response was observed with antagonistic properties at lower concentrations and agonistic effects at higher concentrations tested. The demonstrated androgenic properties of the higher concentrations demonstrate that further investigations should focus on the safety as well as on potential anabolic effects of 5α-hydroxy-laxogenin. This is of interest with regard to abuse for doping purposes.

## Introduction

The World Anti-Doping Agency (WADA) publishes annually the lists of substances and methods prohibited as doping (World Anti-Doping Agency 2022). Prerequisite for including a substance or method is the fulfillment of two out of three criteria defined by the WADA. Those are the medical or scientific evidences that a substance or method possesses either sport performance enhancing effects, (potential) health risk to athletes or violates the spirit of sport (World Anti-Doping Agency 2021).

Dietary supplements might contain illegally added compounds with performance enhancing effects, which, for instance, are published by the U.S. Food and Drug Administration on its Dietary Supplement Ingredient Advisory List (U.S. Food and Drug Administration 2021). Among them, 5α-hydroxy-laxogenin, a derivative of the spirostane-type steroid laxogenin is advertised as plant-based anabolic agent. Whereas the natural occurrence has been shown for laxogenin in several smilax species as well as in two *Allium* species (Akahori and Yasuda 1963; Kim et al. 1991; Kubo et al. 1992; Baba et al. 2000; Timité et al. 2013), there is no proof of a natural existence of 5α-hydroxy-laxogenin. In contrast, Avula et al. recently showed the synthetic origin of 5α-hydroxy-laxogenin detected in dietary supplements (Avula et al. 2019). Since 2019, 5α-hydroxy-laxogenin is included in the FDA list and proven to be contained in dietary supplements (Cohen et al. 2020), but data on potential anabolic effects are missing.

The intention of the present study was to get a first insight on potential androgenic properties of 5α-hydroxy-laxogenin. Therefore, androgen receptor transactivation was investigated in vitro in the yeast androgen screen as well as in a reporter gene assay in a human prostate cell line.

## Materials and methods

Sigma-Aldrich (Munich, Germany) provided dihydrotestosterone (DHT, purity ≥ 97.5%), bicalutamide (purity ≥ 98%) and hydroxyflutamide (OHF, purity ≥ 98%). 5α-hydroxy-laxogenin (5α-OH-laxogenin, purity > 95%) was purchased from Biomol (Hamburg, Germany). Carl Roth (Karlsruhe, Germany) provided dimethyl sulfoxide (DMSO, purity ≥ 99.5%). All test compounds were dissolved in DMSO. Chlorophenol red-β-D-galactopyranoside was purchased by Roche (Mannheim, Germany). Dulbecco's Modified Eagle’s Medium (DMEM/F12), fetal bovine serum (FBS) and penicillin/streptomycin (P/S) were supplied by BioWest (Nuaillé, France). Qiagen (Hilden, Germany) provided Attractene Transfection Reagent. G418 was supplied by Calbiochem, Merck (Darmstadt, Germany). Luciferase assay system was supplied by Promega (Mannheim, Germany). AppliChem (Darmstadt, Germany) provided bovine serum albumin fraction V (BSA). Dr. Aria Baniahmad (Institute for Human Genetics, University Hospital Jena) kindly provided human PC3(AR)_2_ cells and the reporter plasmid encoding the luciferase gene controlled by the mouse mammary tumor virus long repeat promotor (mmTV-luc).

Yeast androgen screen was performed using a *Saccharomyces cerevisiae* strain stably transfected with a human androgen receptor (AR) construct and a reporter plasmid carrying the β-galactosidase encoding *LacZ* gene under the control of androgen responsive elements. Yeast cells were either treated with DMSO as solvent control, DHT at concentrations of 10^–11^ M to 10^–6^ M as positive control or serial dilutions of 5α-OH-laxogenin in the range from 0.01 µg/mL to 100 µg/mL (2.2 × 10^–8^ M to 2.2 × 10^–4^ M respectively). For antagonization, 5α-OH-laxogenin was co-incubated with 10^–5^ M OH-flutamide. β-galactosidase activity was measured by hydrolysis of chloro-phenol red-β-D-galactopyranoside at 565 nm and corrected by the optical density at 690 nm respectively. All treatments were performed in three independent experiments. Statistical significance was assessed by Student’s *t* test considering *p* < 0.05 as significant.

Human PC3(AR)_2_ cells were maintained in DMEM/F12 supplemented with 1% P/S, 0.25 mg/mL G418 and 10% FBS. For experiments, PC3(AR)_2_ cells were cultivated in DMEM/F12 containing 1% P/S and 5% dextran-coated charcoal-treated FBS. 70,000 cells seeded per well in a 24-well plate were transiently transfected with the reporter plasmid mmTV-luc (0.2 µg plasmid DNA and 0.5 µL Attractene per well). Cells were either treated with 0.1% DMSO as solvent control, 5 × 10^–9^ M DHT as positive control or 5α-OH-laxogenin, respectively (final concentration of DMSO was kept at 0.1% in every treatment). For antagonization experiments, cells were co-incubated with 5 × 10^–7^ M bicalutamide and 50 µg/mL 5α-OH-laxogenin. According to the manufacturer’s protocol, we measured luciferase activity and quantified protein concentration using the bicinchoninic acid assay with BSA as standard protein. We calculated Relative Luminescence Units (RLU) by normalizing the luminescence with the protein concentration. Three independent cell culture experiments were performed from which means ± standard deviation are shown. Statistical significance was assessed by one-way ANOVA followed by Bonferroni’s post hoc test, *p* < 0.05 as significant.

## Results and discussion

**Fig. 1:**
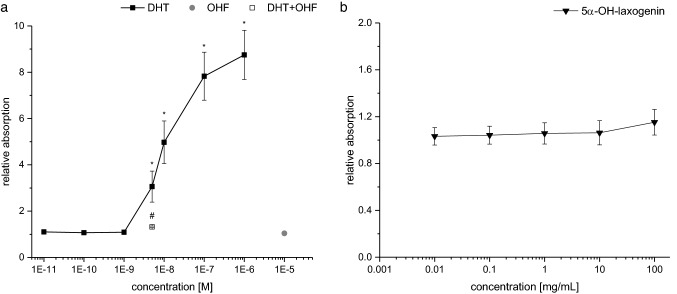
Androgenic dose response in the yeast androgen screen. **a** As positive control, dihydrotestosterone was used at concentrations of 10^–11^ M to 10^–6^ M (DHT, closed square). Reporter gene expression induced by 5 × 10^–9^ M DHT was antagonized with co-incubation with 10^–5^ M OH-flutamide (DHT + OHF, open square). **b** 5α-hydroxy-laxogenin was used at concentrations of 0.01 µg/mL to 100 µg/mL (5α-OH-laxogenin, closed inverted triangle). Relative absorption was normalized to DMSO as solvent control (DMSO = 1). Statistical significance was tested by Student’s *t* test. *Denotes statistically significant differences compared to the solvent control (DMSO = 100%, *p* < 0.05). ^#^Denotes statistically significant differences compared to the 5 × 10^–9^ M DHT incubation (*p* < 0.05)

In the yeast androgen screen, DHT stimulated the reporter gene expression in a dose-dependent manner (Fig. 1a). However, none of the 5α-hydroxy-laxogenin concentrations induced the reporter gene β-galactosidase (Fig. 1b).

**Fig. 2:**
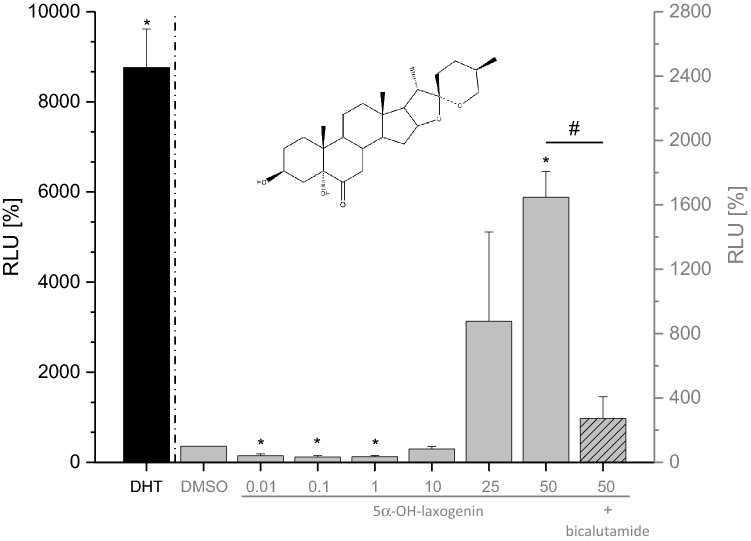
Androgen receptor transactivation in human PC3(AR)_2_ cells. Luciferase activity is shown in response to 5 × 10^–9^ M DHT as positive control (black column) or to 5α-OH-laxogenin (structure shown) at increasing concentrations of 0.01 µg/mL to 50 mg/mL (gray columns). Co-incubation with 5 × 10^–7^ M bicalutamide antagonized luciferase expression induced by 50 µg/mL 5α-OH-laxogenin. Statistical significance was tested by one-way ANOVA followed by Bonferroni’s post hoc test. *Denotes statistically significant differences compared to the solvent control (DMSO = 100%, *p* < 0.05). ^#^Denotes statistically significant differences compared to the 50 µg/mL 5α-OH-laxogenin incubation (*p* < 0.05)

In contrast, 5α-hydroxy-laxogenin was able to induce the luciferase expression in human PC3(AR)_2_ cells in a biphasic dose-dependent manner, with antagonistic effects at lower doses (0.01–1 µg/mL) and agonistic effects at higher doses (Fig. 2). Co-incubation with bicalutamide, a non-steroidal AR antagonist, antagonized the 5α-hydroxy-laxogenin-induced luciferase activity (Fig. 2). This clearly demonstrated that 5α-hydroxy-laxogenin binds to the human AR and acts as an agonist in the PC3(AR)_2_ cells at higher doses. The discrepancy between the two bioassays might be due to the different co-factor pattern in yeast and mammalian cells as well as the additional yeast cell wall, which might prevent diffusion. Hence, false negative results in the yeast androgen screen of substances showing clear androgenic properties in mammalian cells are possible, e.g., shown for *p,p*’-DDE by Gaido and colleagues (Gaido et al. 1997; Endocrine Disruptor Screening and Testing Advisory Committee 1998).

The observed androgenic potential of higher 5α-hydroxy-laxogenin concentrations observed herein raises possible safety concerns regarding reproductive organs (e.g., prostate). Besides the androgenic activity, further investigations should focus on potential anabolic activities of 5α-hydroxy-laxogenin. The proof of anabolic effects would meet WADA’s criterion on performance enhancement. Moreover, data on pharmacokinetics as well as on biotransformation should be gathered to contribute to an estimation of the efficacy and safety of 5α-hydroxy-laxogenin.

In conclusion, we showed androgenic potential of 5α-hydroxy-laxogenin in an in vitro bioassay for the first time. As this synthetic spirostane-type steroid is marketed as dietary supplement for athletes, future investigations focusing on potential anabolic properties and safety of 5α-hydroxy-laxogenin intake are necessary.
